# Association of Biomarker Level with Cardiovascular Events: Results of a 4-Year Follow-Up Study

**DOI:** 10.1155/2020/8020674

**Published:** 2020-06-23

**Authors:** L. Turgunova, B. Baidildina, Y. Laryushina, B. Koichubekov, A. Turmukhambetova, L. Akhmaltdinova

**Affiliations:** Karaganda Medical University, Karaganda, Kazakhstan

## Abstract

**Background:**

Given the high rates of morbidity and mortality from cardiovascular disease (CVD), the primary and secondary CVD prevention is one of the public health priority. Inflammation and endothelial dysfunction are the major drivers of atherosclerosis development and progression. In this regard, the study of the biomarker application as a tool to better identify high-risk individuals is an up-to-date sector of modern cardiology. The simultaneous measurement of multiple biomarkers can increase the risk stratification for people who are not known to have cardiovascular events in their history. The study aimed to investigate the predictive value of chemokine (C-X-C motif) ligand 16 (CXCL16), endocan, and heart-type fatty acid binding protein (H-FABP) in the cardiovascular event development in people who are not known to have cardiovascular events in their history.

**Method:**

We examined 363 people aged 30 to 65 who have been living permanently in the city of Saran, Karaganda region. The selected participants were people registered at a clinic at the city of Saran, who were screened between August and September 2014.

**Results:**

The follow-up period was 48 months (from August-September 2014 to November 2018). The results showed that CXCL16 (*p* < 0.001), endocan (*p* < 0.001), and H-FABP (*p*=0.002) biomarker levels are significantly higher in outcome groups compared with those of the no-outcome group. Univariate regression analysis proved the prognostic significance of all biomarkers in cardiovascular events development. The multivariate regression analysis after the adjustment confirmed that the CXCL16 increase was associated with the “composite endpoint” (CE) development (*p* < 0.001) while the endocan increased due to the development of major cardiovascular events (MACE) (*p*=0.008); we did not find the association of the risks of event development with the H-FABP level increase (*p*=0.83).

## 1. Introduction

The morbidity due to CVD is very high, thus making them the leading global cause of death. The cardiovascular diseases represent 13.0–15.3% of the total morbidity and 24.4–25.9% of the mortality causes in the Republic of Kazakhstan [1].

Atherosclerosis, as the cause of fatal events development, is a long-term and complex multifactorial process. Such traditional risk factors for CVD as high cholesterol, hypertension, smoking, and diabetes mellitus type 2 (DM 2) have been well known for decades and were included in the predictive models. Early detection of individuals at risk of cardiovascular events has enabled preventive services, which reduced the rate of CVD mortality and disability. However, up to 20% of patients with coronary heart disease (CHD) do not have traditional risk factors, while 40% have only a single one [2].

The recent decades have seen the rise in the prognostic value of biomarkers taking into account the significant role of endothelial dysfunction and systemic inflammation in the progression and destabilization of atherosclerotic plaques [3].

CXCL16, as a proinflammatory cytokine, is closely associated with atherosclerosis incidence. CXCL16 promotes the T-lymphocyte adhesion to endothelium and the T-cell migration to the inflammation site. It functions as a scavenger receptor and stimulates the low-density lipoproteins (LDL) to be absorbed by macrophages. Wang et al. confirm the high level of CXCL16 expression in atherosclerotic plaques, which increases the level of interferon gamma (INF-*γ*) and can contribute to the transformation of a stable plaque into a vulnerable one [4]. High levels of soluble CXCL16 during acute cardiovascular events may indicate a failure of long-term prediction, but nobody knows whether the CXCL16 is associated with the CVD risk in healthy people. One study has shown that among patients with no previous incidence of coronary events, those with a high level of CXCL16 had twice the risk of heart attack (HA) exposure [5].

Endoсan (formerly known as an endothelial cell-specific molecule-1, ESM-1) can stimulate the endothelial cells to produce more types of inflammatory cytokines, increase the vascular permeability, and stimulate the leukocyte migration, which plays a vital role in the pathogenesis of various phases of atherosclerosis. High serum endocan levels may be one of the independent risk factors for acute coronary syndrome (ACS) [6]. They have found the correlations between serum endocan levels and subclinical atherosclerosis in patients with DM 2. The facts suggest that serum endocan levels can be a useful biomarker for early diagnosis of subclinical atherosclerosis in patients with DM 2 [7]. Monitoring of serum endocan levels can be an essential step for future prediction of the disease onset and progression [8]. With regard to patients with chronic kidney disease (CKD), the addition of endocan levels to the prognostic model with traditional risk factors has improved the prediction of lethal and nonlethal cardiovascular events combined with traditional risk factors [9]. We have not found prospective studies on the association of the endocan level among people who are not known to have cardiovascular events in history.

There are several sensitive markers for myocardial damage. Some studies prove the predictive value of the H-FABP level for cardiovascular (CV) events in patients with stable angina [10]. Higher H-FABP levels in patients with cardiovascular risk factors such as hypertension, insulin resistance, diabetes mellitus, obesity, metabolic syndrome, and chronic kidney disease were independent predictors of all CV events among high-risk subjects in the general population [11]. Studies in patients with essential hypertension have proven that the H-FABP level is a new and useful marker for CV events prediction in the case of hypertension [12].

The simultaneous measurement of multiple biomarkers can increase risk stratification for people who are not known to have cardiovascular events in history. There is no recorded prospective study of the predictive value of the chemokine CXCL16, endothelial dysfunction marker endocan, and myocardial injury marker H-FABP in this population.

The study was aimed to investigate the predictive value of chemokine 16, endocan, and H-FABP levels in the development of CV events among people who are not known to have cardiovascular events in their history.

## 2. Materials and Methods

Baseline characteristics of the examined subjects: we examined 363 people aged 30 to 65 who have been living permanently in the city of Saran, Karaganda region. The selected participants were people registered at a clinic of the city of Saran, who were screened between August and September 2014 [13]. The exclusion criteria were pregnancy and clinical manifestations of cardiac decompensation. All participants submitted an informed consent to participate in the study. During the study, we also used the questionnaire developed for the study participant. The questionnaire was comprised of questions about gender, age, social factors (income level, marital status, and level), presence or absence of myocardial infarction (MI), chronic CHD, peripheral artery atherosclerosis, DM 2 in the subject's history, presence or absence of a positive family history of CVD, whether they get a 30-minute physical activity (PA) per day or not, and whether they were active smokers or not. All patients were diagnosed with hypertension (HTN). The blood pressure was measured according to European guidelines [14]. The body mass index (BMI) was defined as a person's weight (in kg) divided by the square of the person's height (m2). BMI from 18.5 to 24.9 kg/m^2^ was defined as normal, from 25 to 30 kg/m^2^ as overweight, and ≥30 kg/m^2^ as obesity [15]. All patients were tested for glucose and cholesterol levels. The capillary blood glucose level was measured by an electrochemical method using an Accu-Chek Active by Akku-Chek blood glucose meter. The DM was considered to be verified when there were criteria which the World Health Organization (WHO) set in 2001: glucose concentration in whole blood is >6.1 mmol/l, and glucose concentration is >11.1 mmol/l in random measurement. Plasma for the study was obtained via the standard phlebotomy technique from ethylenediaminetetraacetic acid (EDTA) anticoagulants. Plasma was aliquoted into cryovials and got frozen quickly. The samples were stored in a low-temperature refrigerator (−70°C) until the study commencement (up to 3 months). CXCL16, H-FABP, endocan were measured by magnetic bead-based multiplex immunofluorescence assay through the Xmap technology using the Milliplex map Human CVD Magnetic Bead Panel 1 (Millipore). The study was conducted under the manufacturer's instructions using the Override Protocol. The method is based on incubation of unknown, standard and control samples with magnetic beads conjugated with primary antibodies. After washes, the detection was performed using detecting antibodies, streptavidin-phycoerythrin, and the final stage was fluorescence recording on the Bioplex 3D system (Luminex software). Minimum detectable concentrations were as follows: CXCL16-13.2 pg/mL; endocan-11.5 pg/mL; H-FABP-24.9 pg/mL. The intraassay % coefficient of variation (CV) was less than 20% for all analytes.

The follow-up period was 48 months (from August-September 2014 to November 2018). Subjects were monitored via the telephone and through information collected from the medical information system, which contains records of all treatment and/or hospitalization instances of patients. The composite endpoint included the presence of any of the following events: cardiovascular death, MI, stroke, hospitalization and/or application due to CHD, and transient ischemic attack. A MACE group comprised of patients with nonfatal MI, nonfatal stroke, and cardiovascular death was distinguished from this group. The stroke was defined as a sudden onset of nonconvulsive and focal neurological deficit due to ischemia or hemorrhage that lasts >24 hours, and it is transient ischemic attack if neurological recovery occurred within 24 hours. The diagnosis of stroke and the transient ischemic attack was based on the disease history, neurological examination, and all available clinical data, including computed tomography (CT)/magnetic resonance imaging (MRI) results. Coronary heart disease included myocardial infarction, sudden cardiac death, and newly diagnosed angina. The criteria for MI were at least two of the following: (1) typical symptoms, including prolonged severe anterior chest pain; (2) elevated cardiac enzymes—higher than twice the upper limit of the standard values; sudden cardiac death within 1 hour of the acute condition onset; (3) progressive diagnostic electrocardiographic changes. The cases of newly diagnosed angina were determined based on typical angina-type pain, reversed by the use of nitrates, confirmed by a doctor's examination, and via the electrocardiography (ECG) exercise test. Out of 363 patients, 52 were excluded to follow up because they noted the history of stroke or angina. Other 44 patients have also been lost to follow-up for various reasons: the development of cancer (*n* = 4), those with incomplete data (*n* = 24), and the change of residence (*n* = 16); thus, the final sample comprised of 267 people.

### 2.1. Statistical Analysis

Statistical data processing was performed using SPSS (version 22.0). Quantitative variables in normal distribution were represented as mean values and their standard deviations (M ± SD); if they were different from the normal distribution they were represented as a median and interquartile range (Me (IQR). Dichotomies were represented as fractions (absolute number of patients (%). Age, education background and income, systolic (SBP), diastolic blood pressure (DBP), BMI, waist length (WL), and Systematic COronary Risk Evaluation (SCORE) risk were used as categorical variables. We distinguished 25–44, 45–59, 60 years, and older age groups. As regards the educational level, the respondents were distinguished by those who have secondary or lower-level education, vocational secondary and higher education; by income level as lower-than-middle and low, middle, higher-than-middle, and high. Using the SCORE scale, we distinguished groups with low risk (less than 1%), moderate (1–5%), and high and very high (5% or more). Sex, smoking, waist length, cholesterol, and glucose levels were used as dichotomous variables. We used the Mann–Whitney *U* test in descriptive statistics for independent samples to compare quantitative data; the categorical data were analyzed using the *χ*^2^ Pearson test. The Spearman correlation analysis (Rho) was used to determine the relationship between the parameters. Using a single-factor and multifactor COX regression, we analyzed the association of the CXCL16, endocan, and H-FABP levels with the developments of the composite endpoint and acute cardiovascular events and set independent predictors and odds ratios (OR) at 95% confidence interval (CI) for each factor. The multiple factor analysis included the covariates with *p* < 0.05 or if they changed the value of the main effect by≥10%. Given the strong correlation between BMI and WL (*r* = 0.799), the BMI^*∗*^C variable, which shows the relationship between the predictors, was included in the multiple factor analysis. During the hypothesis test, we applied a critical level of significance of*p* of 0.05; for multiple comparisons, we used a statistically significant level of *p*=0.025.

## 3. Results

During the 4-year follow-up period, 43 (16.1%) of respondents had a composite endpoint, of which 15 (5.6%) had MACE,4 MI, 6 stroke, 5 cardiac death, 6 transient ischemic attack, and 22 newly diagnosed angina cases.  Table 1 gives the characteristics of the survey. The average age of respondents was 53 ± 10 years and was significantly higher in groups with an event. Groups with and without event had not been distinguished by sex, education level, income level, and marital status (Table 1). Most of the respondents had secondary and vocational secondary education (37% and 45%, respectively) and were married; 72% said their income level was of a middle and below-middle level. When comparing the two groups, we did not find significant differences between smoking rates, DBP levels, and total cholesterol. Still, SBP level, BMI, and frequency of abdominal obesity (AO) were significantly different between groups (Table 1). The percentage of smokers among respondents was low and amounted to 9%, which could affect the assessment of its significance.

Table 2 shows the results of the biochemical tests in groups with or without an outcome. The comparison of biochemical parameters between groups showed that the glucose level of 5.85 (Q1–Q3 5, 10–5, 80; *p*=0.21) and cholesterol level of 5.56 (Q1–Q3 4, 68–6, 33; *p*=0.56) in the no-outcome group was not significantly different as compared with the “composite endpoint” groups: glucose level of 6.32 (Q1–Q3 5.10–6.1) and cholesterol level of 5.66 (Q1–Q3 4.50–6.59) and with MACE group: 6.92 (Q1–Q3 5.20–6.30) and 5.53 (Q1–Q3 4.37–7.08), respectively, despite the blood glucose upward tendency in the event groups. Comparative analysis of biochemical markers between groups showed that the levels of CXCL16, endocan, and H-FABP were significantly higher in the “composite endpoint” group and in the MACE group as compared with those of the no-event group.

The correlation analysis showed the absence of significant interrelations of CXCL16 and endocan with BMI, WL, SBP, DBP, cholesterol level, blood glucose, and smoking (Table 3), while we noted a weak direct correlation dependence of CXCL16 with sex of endocan with the respondents' age. H-FABP showed the most considerable number of correlations; its level was higher depending on the age, BMI, presence of AO, and blood glucose level.

The results of the unadjusted hazard ratio for variables showed that CXCL16 and endocan are of predictive value in the development of the “composite endpoint” and MACE (Table 4). After adjusting the variables (i.e., age, sex, BMI, smoking, hypertension, glycemia, and cholesterol), the endocan level was not associated with the development of the composite endpoint. Still, the raise of CXCL16 level was a significant predictor both for the development of the “composite endpoint” and MACE after the adjustments in multivariate analysis. We have not found the H-FABP level associated with the development of cardiovascular events during the follow-up period.

## 4. Discussion

Our results showed that the predictive value of CXCL16, endocan, and H-FABP is different in the development of cardiovascular events in a group of people who are not known to have CV events. After the adjustment in the multidimensional regression analysis, the increase in the chemokine level was associated with the development of the “composite endpoint,” and increase in the the endocan level with the development of a MACE; we did not find an association between the risk of events and H-FABP level.

CXCL16 plays a significant role in atherosclerosis development and progression [8–11]. Yi G. W. found the close relationship between the serum soluble concentration of CXCL16 and the severity of coronary atherosclerotic heart disease and the degree of coronary artery stenosis [16]. Smith et al. reported increased plasma levels of CXCL16 in CHD independent of co-morality such as DM 2 and HTN [17]. The association of CXCL16 with the development of cardiovascular diseases was a topic for discussion in various studies. CXCL16 level raise was an independent predictor of adverse outcomes in patients with MI after percutaneous coronary intervention in hemodialyzed patients [18]. CXCL16 level raise was associated with the development of ischemic stroke caused by atherosclerotic occlusion of carotids. The authors found that the CXCL16 level in serum rises upon the increase of the plaque and lumen stenosis area and has no significant correlation with risk factors such as sex, age, family history, smoking, BMI, blood glucose and lipids, mean SBP, and DBP [19]. Given that the listed risk factors were closely related to stroke, the authors supposed that the role of a high CXCL16 in stroke was mainly mediated through an inflammatory response and contributed to the plaque formation and rupture. The discovered dependence of the chemokine level on the microembolic signals (MES), which are considered evidence of plaque destabilization, indicates the participation of chemokine in the plaque destabilization and says that chemokine can be an independent predictor of the atherosclerotic ischemic stroke development. We found one prospective study that showed a positive CXCL16 association with the risk of MI development among people who are not known to have cardiovascular events. The authors also revealed the absence of significant correlations between the chemokine level and BMI, total cholesterol, smoking, HTN, DM 2, creatinine level, or high-sensitivity C-reactive protein (CRP). CXCL16 functions have not been fully defined and differ in membrane-associated and soluble forms. Given membrane-associated CXCL16 forms can mediate the absorption and release of oxidized LDL [19, 20], preventing the development of atherosclerosis plaque, the soluble forms can cause inflammatory reactions in smooth muscle cells of vessels and peripheral blood mononuclear cells, giving a proatherosclerotic effect [16, 17]. The participation of inflammatory cytokines such as tumor necrosis factor (TNF), interleukin (IL)-1b, and IFN-*γ* at all stages of atherosclerosis is confirmed in some experimental works. It is assumed that chemokine can be a reliable biomarker for atherosclerotic disorders due to its ability to split off the soluble CXCL16 from its membrane-associated form as a result of TNF, IL-1b, and IFN-*γ* activation and thus activating the inflammatory cascade at the level of smooth muscle cells of vessels, causing the progression and destabilization of atherosclerotic plaques.

Endocan is secreted by endothelial cells of vessels and plays a key role in endothelial dysfunction and inflammatory reactions [6, 8]. Kundi et al. have shown that the endocan level can predict the MI with ST-segment elevation and is an independent predictor of adverse outcomes in patients with MI [21]. Other authors have found that endocan levels have a predictive value in major cardiovascular events development in patients with stress hyperglycemia and the MI with ST-segment elevation; endocan levels were independent prognostic factors in both fatal and nonfatal cardiovascular events in patients with CKD [9]. We have not found studies investigating the association of endocan with the CV event development in people who are not known to have cardiovascular events. The results of our study showed that endocan levels are significantly higher in people with events compared with those of the group without events and have an association with the development of major cardiovascular events. Several growth actors and cytokines regulate the security of the endocan. IL-1, TNF, and lipopolysaccharide stimulate in the in vitro expression of endocan. Endocan affects the interaction of the intercellular adhesion molecules-1/LFA-1, which facilitates the recruitment and accumulation of leukocytes in vascular endothelium and thus can be involved in the control of leukocyte extravasation in inflammation sites [20]. In patients with CKD, the confirmed correlation of high serum endocan levels with other inflammation parameters, such as the number of leukocytes, neutrophils, and platelets, proving its role in systemic inflammation.

We found that the H-FABP level was significantly higher in people with cardiovascular events, but H-FABP was not found prognostic in people who had no history of cardiovascular events. As some studies showed, a raised H-FABP level, indicating a latent myocardial damage, is associated with arterial hypertension, insulin resistance, diabetes mellitus, metabolic syndrome, and chronic kidney disease [22]. It is noteworthy that H-FABP was reported to have been found in all members of the overall population, which indicated a latent myocardial damage in the overall population. It is reported that H-FABP is an independent predictor of death for all causes, including CVDs. The higher H-ABP levels were associated with an increase in cardiovascular risk factors, which are the triggers for ongoing myocardial damage worsening. These data are consistent with our study, where we found the direct significant H-FABP level relationships with the glucose level, BMI, and abdominal obesity. Most previous review articles discussed the diagnostic role of H-FABP in conditions such as ACS and acute pulmonary embolism and predictive role in patients with stable angina [10], arrhythmias, heart failure, and MI. A specific clinical situation is of great importance for assessing myocardial damage since cardiovascular diseases develop due to complex physiological and pathophysiology processes associated with genetic and cardiovascular risk factors [23]. We believe that the absence of predictive value of H-FABP in our study is due to some extent to the high percentage of persons with BMI ≥ 25 kg/m^2^ (65%), AO (39%), and the presence of HTN in the group without the development of cardiovascular events.

## 5. Restrictions

The main limitation of our study was the small number of people with an event, which is primarily associated with the inclusion of persons up to 65 y.o. without cardiovascular events in history. This group of individuals requires a longer follow-up period to increase the number of findings. Another limitation was the prevalence of female, a large number of persons who refused to be followed up or were excluded from the study. We did not make a comparative assessment of the predictive value of biomarkers with accepted cardiovascular risk scales. In Kazakhstan, a screening procedure is carried out under the SCORE scale, which estimates a 10-year risk. Despite the heterogeneity of patients and the small cohort, we managed to identify an association of biomarker with the development of cardiovascular events. Differences in the predictive values of individual biomarkers justify the need for a differentiated approach to their estimation and application. We need further prospective observations to explore the possibility of better stratification of cardiovascular risk groups among those who are not known to have cardiovascular events in history.

## 6. Conclusion

The results showed that CXCL-16, endocan, and H-FABP biomarker levels are significantly higher in outcome groups compared with those of the no-outcome group. Univariate regression analysis proved the prognostic significance of all biomarkers in cardiovascular event development. These data indicate that markers of inflammation, endothelial dysfunction, and lesion of target organs play an essential role in the development of the CV event. After the adjustment in the multivariate regression analysis, we found that the chemokine level raise was associated with the development of a “composite endpoint,” and the endocan level raise was associated with the development of major cardiovascular events; we did not find an association of the CV event risk development with H-FABP level increase. Contrary to other biomarkers, the H-FABP level was more influenced by age, BMI, AO, and blood glucose level. We need further prospective studies to investigate the predictive value of biomarkers in people who are not known to have cardiovascular events in history and estimate the additional benefit of their application compared with well-known forecasting scales.

## Figures and Tables

**Table 1:** Clinical characterization of patients in the no-outcome group and in the outcome group (composite endpoint) and with MACE.

Variables	Total *n* = 267	No outcome *n* = 224	Composite end point *n* = 43	MACE *n* = 15	*χ* ^2^^*∗*^	*p* level^*∗*^	*χ* ^2^^*∗∗*^	*p* level^*∗∗*^
Sex, *n* (%)					0.41	0.68	1.52	0.13
Female, *n* (%)	175 (65.5)	148 (66.1)	27 (62.8)	7 (46.7)				
Male, *n* (%)	92 (34.5)	76 (33.9)	16 (37.2)	8 (53.8)				
Age, years (Q25–Q75)	54 (45–59)	53 (44–58)	56 (52–63)	58.5 (53–61)	**3.23**	**≤0.001**	1.82	0.069
30–44, *n* (%)	62 (23.2)	60 (26.8)	2 (4.7)	—				
45–59, *n* (%)	141 (52.8)	118 (52.7)	23 (53.5)	10 (66.7)				
>60, *n* (%)	64 (24.0)	46 (20.5)	18 (41.9)	5 (33.3)				
Educational background, *n* (%)					0.67	0.50	0.39	0.70
Secondary and lower level, *n* (%)	97 (36.3)	85 (37.9)	12 (27.9)	6 (40.0)				
Vocational secondary, *n* (%)	119 (52.8)	95 (42.4)	24 (55.8)	7 (46.7)				
Higher, *n* (%)	51 (24.0)	44 (19.6)	7 (16.3)	2 (13.3)				
Marital status, *n* (%)					0.70	0.49	0.38	0.18
Married, *n* (%)	174 (65.2)	148 (66.1)	26 (60.5)	11 (73.3)				
Not married, *n* (%)	22 (8.2)	18 (8.0)	4 (9.3)	1 (6.7)				
Divorced/widowed, *n* (%)	71 (26.6)	54 (25.9)	13 (30.2)	3 (20.0)				
Income level, *n* (%)					1.08	0.28	1.33	0.18
Lower than middle and low, *n* (%)	131 (49.1)	111 (49.6)	20 (46.5)	6 (40.0)				
Middle, *n* (%)	91 (34.1)	77 (34.4)	14 (32.6)	6 (40.0)				
Above middle and high, *n* (%)	33 (12.4)	25 (11.2)	8 (18.6)	3 (20.0)				
No answer, *n* (%)	12 (4.5)	11 (4.9)	1 (2.3)	—				
BMI, kg/m^2^, median (Q25–Q75)	28.1 (24.8–32.3)	28 (24.6–31)	32.2 (25.5–38.6)	30.3 (27–34)	**2.93**	**0.003**	1.91	0.056
Overweight (BMI 25–29.9 kg/m^2^), *n* (%)	105 (39.3%)	92 (41.1%)	13 (30.2%)	2 (13.3)				
Obesity (BMI ≥ 30 kg/m2), *n* (%)	91 (34.1%)	66 (29.5%)	25 (58.1%)	10 (66.7)				
Waist length, cm, median (Q25–Q75)	96 (86–104)	95 (86–103)	108 (96–115)	95.5 (85.2–108.7)	**2.26**	**0.024**	**3.01**	**0.003**
The presence of AO, *n* (%)	195 (73%)	160 (71.4%)	35 (81.4%)	14 (93.3)				
SBP (mm hg), median (Q25–Q75)	130 (120–140	130 (117.5–140)	140 (140–170)	130 (120–150)	**2.28**	**0.022**	**3.30**	**≤0.001**
DBP (mm hg), median (Q25–Q75)	80 (80–90)	80 (80–90)	90 (80–100)	80 (72–90)	1.25	0.21	**2.57**	**0.01**
Smoking (%)	30 (11.2)	28 (12.5)	2 (4.7)	2 (13.3)	1.49	0.14	0.09	0.93

^*∗*^ *χ* ^2^ and *p* between the no-outcome group and outcome group with a composite endpoint; ^*∗∗*^*χ*^2^ and *p* between the no-outcome group and outcome group with a MACE.

**Table 2:** Level of biochemical parameters in groups with and without event.

Variables	Total *N* = 223	No outcome *N* = 188 (84.3%)	Composite end point *N* = 35 (15.7%)	MACE *n* = 15	*χ* ^2^^*∗*^	*p* level^*∗*^	*χ* ^2^^*∗∗*^	*p* level^*∗∗*^
Cholesterol, mmol/L, median (Q25–Q75)	5.3 (4.57–6.3)	5.56 (4.68–6.33)	5.66 (4.50–6.59)	5.53 (4.37–7.08)	0.59	0.56	0.09	0.93
Glucose, mmol/L, median (Q25–Q75)	5.4 (5.1–5.8)	5.85 (5.10–5.80)	6.32 (5.10–6.1)	6.92 (5.20–6.30)	1.56	0.21	0.88	0.34
Endocan, pg/ml, median (Q25–Q75)	733.2 (68.8–3432.8)	718.6 (101.8–3432.8)	957.0 (68.8–2150.9)	1185.6 (355.1–2150.9)	2.39	0.017	3.27	≤0.001
CXCL16, pg/ml, median (Q25–Q75)	324.8 (4–2673.0)	310.0 (4–1685)	432.3 (64–2673.0)	774.2 (293.0–2673.0)	2.62	0.009	3.45	≤0.001
H-FABP, pg/ml, median (Q25–Q75)	2346.8 (343.2–53447.7)	2154.2 (422.4–53447.7)	3245.6 (343.2–23509.9)	5443.4 (1837.4–23509.9)	2.87	0.004	3.10	0.002

^*∗*^ *χ* ^2^ and *p* between the no-outcome group and outcome group with a composite endpoint; ^*∗∗*^*χ*^2^ and *p* between the no-outcome group and outcome group with a MACE.

**Table 3:** Correlation between the level of CXCL16, endocan, and H-FABP with the clinical laboratory characteristics of respondents.

Variables	CXCL16		H-FABP		Endocan	
	*r*	*p*	*r*	*p*	*r*	*p*
Age, years	0.04	0.51	**0.18** ^*∗*^	**0.007**	**0.13** ^*∗*^	**0.046**
Sex	**0.19** ^*∗∗*^	**0.004**	0.09	0.160	0.06	0.382
BMI, kg/m^2^	0.01	0.88	**0.15** ^*∗*^	**0.023**	0.03	0.635
Waist length, cm	0.02	0.73	**0.23** ^*∗∗*^	**≤0.001**	0.07	0.304
SBP, mm Hg	0.04	0.51	0.11	0.112	0.11	0.110
DBP, mm Hg	0.05	0.41	0.09	0.153	0.11	0.113
Glucose, mmol/l	0.01	0.81	**0.31** ^*∗∗*^	**≤0.001**	0.09	0.194
Cholesterol, mmol/l	0.05	0.39	0.08	0.228	0.02	0.110
Smoking	−0.01	0.86	0.02	0.723	0.09	0.180

^*∗*^Correlation is significant at 0.05 (two-tailed); ^*∗∗*^correlation is significant at 0.01 (two-tailed).

**Table 4:** Results of a single-factor and multiple-factor COX regression analysis to estimate risk factors of a “composite endpoint” among people who are not known to have cardiovascular events.

Variable	cOR (95% CI)	*p*	aOR^*∗*^ (95% CI)	*p*
Composite endpoint^*∗*^ (EP)				
Age, years	**1.07 (1.02–1.12)**	**0.004** ^*∗*^	**1.06 (1.006–1.11)**	**0.028** ^*∗*^
Gender, male	1.16 (0.53–2.56)	0.71	1.72 (0.70–4.19)	0.237
Smoking	0.29 (0.04–2.14)	0.227	0.27 (0.03–2.18)	0.218
BMI, kg/m^2^	1.07 (1.007–1.13)	0.027	—	—
WL, cm	1.025 (1.002–1.050)	0.035	—	—
BMI ∗ WM, kg/m^2^^*∗*^cm	—	—	1.001 (1.000–1.001)	0.084
SBP, mmHg	**2.09 (1.07–4.07)**	**0.031** ^*∗*^	**1.03 (1.005–1.06)**	**0.022** ^*∗*^
DBP, mmHg	1.47 (0.76–2.85)	0.25	0.96 (0.91–1.01)	0.113
Cholesterol, mmol/l	0.92 (0.44–1.94)	0.84	0.95 (0.70–1.28)	0.736
Glucose, mmol/l	1.80 (0.93–3.50)	0.082	0.98 (0.85–1.14)	0.845
CXCL16 pg/ml	**1.002 (1.001–1.003)**	**≤0.001** ^*∗*^	**1.002 (1.001–1.003)**	**≤0.001** ^*∗*^
Endocan, pg/ml	**1.001 (1.0–1.001)**	**0.024** ^*∗*^	1.001 (1.000–1.001)	0.370
H-FABP, pg/ml	1.000 (1.000–1.000)	0.455	1.000 (1.000–1.000)	0.756

MACE				
Age, years	1.07 (0.99–1.16)	0.096	1.02 (0.91–1.15)	0.139
Gender, male	1.18 (0.33–4.24)	0.80	5.22 (0.58–46.7)	0.678
Smoking	1.01 (0.13–7.89)	0.993	0.078 (0.001–18.5)	0.361
BMI, kg/m^2^	1.098 (0.99–1.21)	0.073	—	—
WL, cm	1.060 (1.015–1.107)	0.008	—	—
BMI^*∗*^WM, kg/m^2^ ∗ cm	—	—	**1.001 (1.000–1.1002)**	**0.028** ^*∗*^
SBP, mmHg	**1.04 (1.018–1.065)**	**≤0.001** ^*∗*^	**1.10 (1.034–1.18)**	**0.003** ^*∗*^
DBP, mmHg	**1.043 (1.001–1.088)**	**0.045** ^*∗*^	0.916 (0.83–1.02)	0.096
Cholesterol, mmol/l	0.96 (0.58–1.61)	0.881	0.92 (0.92 (0.47–1.83)	0.818
Glucose, mmol/l	1.012 (0.96–1.34)	0.14	0.89 (0.89 (0.67–1.18)	0.415
CXCL16, pg/ml	**1.003 (1.002–1.004)**	**≤0.001** ^*∗*^	**1.004 (1.002–1.006)**	**≤0.001** ^*∗*^
Endocan, pg/ml	**1.001 (1.000–1.002)**	**≤0.001** ^*∗*^	**1.002 (1.001–1.004)**	**0.008** ^*∗*^
H-FABP, pg/ml	1.000 (1.000–1.000)	0.095	1.000 (1.000–1.000)	0.833

## Data Availability

The data used to support the findings of this study are available from the corresponding author upon request.
